# Transcriptomic Responses Induced in Muscle and Adipose Tissues of Growing Pigs by Intravenous Infusion of Sodium Butyrate

**DOI:** 10.3390/biology10060559

**Published:** 2021-06-20

**Authors:** He Zhang, Erdu Ren, Rongying Xu, Yong Su

**Affiliations:** 1 Laboratory of Gastrointestinal Microbiology, Jiangsu Key Laboratory of Gastrointestinal Nutrition and Animal Health, College of Animal Science and Technology, Nanjing Agricultural University, Nanjing 210095, China; 2018205020@njau.edu.cn (H.Z.); 2015105044@njau.edu.cn (E.R.); 2020205027@stu.njau.edu.cn (R.X.); 2 National Center for International Research on Animal Gut Nutrition, Nanjing Agricultural University, Nanjing 210095, China

**Keywords:** adipose, muscle, pig, sodium butyrate, transcriptome

## Abstract

**Simple Summary:**

The present study was constructed to determine the effects of short-term intravenous sodium butyrate (SB) administration on metabolism through transcriptomic responses in muscle and adipose tissue of pigs. We found that systemic butyrate displayed a discriminative metabolic regulation in muscle and adipose tissue. Intravenous SB infusion decreased amino acid metabolism pathways both in muscle and adipose tissues. Intravenous SB infusion increased glucose catabolism in muscle tissue and decreased glucose utilization in adipose tissue. Moreover, intravenous SB infusion decreased lipolysis in muscle tissue but increased lipolysis in adipose tissue. These findings support the direct role of the SCFA butyrate in the regulation of the metabolism.

**Abstract:**

Butyrate has a central function in the regulation of energy metabolism as a metabolite of bacterial fermentation. This study evaluated the effects of intravenous sodium butyrate (SB) administration on the transcriptome of muscle and adipose tissue of pigs. Twelve crossbred barrows (Duroc × Landrace × Large White) were fitted with a medical polyethylene cannula via the internal jugular vein and were daily infused with 10 mL SB (200 mmol/L) or the same volume of physiological saline. Muscle transcriptome showed 11 DEGs related to carbohydrate metabolism, 28 DEGs related to lipid metabolism, and 10 DEGs related to amino acid metabolism. Among these, carbohydrate catabolic process-related genes (*PPP1R3B*, *PRPS2*, *ALDOC*), fatty acid synthase (*FASN*), and lipolysis-related genes (*PLIN1*) were upregulated, while the carbohydrate biosynthetic process-related genes (*PCK1*) and most amino acid metabolism-related genes were downregulated. Adipose transcriptome showed 12 DEGs related to carbohydrate metabolism, 27 DEGs related to lipid metabolism, and 10 DEGs related to amino acid metabolism. Among these, carbohydrate metabolism-related genes (*IGF1*, *LEP*, *SLC2A4*) and lipolysis-related genes (*LPL*) were upregulated, while lipolysis-related genes (*ANGPTL4*) and most amino acid metabolism-related genes were downregulated. The results suggest that short-term intravenous SB infusion could modulate the muscle and adipose tissue metabolism at the transcriptional level by decreasing amino acid metabolism pathways. Additionally, intravenous SB increased the glucose catabolism in muscle tissue and decreased the glucose utilization in adipose tissue. Intravenous SB increased the fatty acid synthesis, decreased the lipolysis in muscle tissue, and increased the lipolysis in adipose tissue. This suggests that systemic butyrate may display discriminative metabolic regulation in different tissues of barrows.

## 1. Introduction

The global incidence of obesity caused by an imbalance between energy intake and expenditure has dramatically increased. Adipose tissue has traditionally been defined as the primary site for lipid storage and mobilization [1] and as an endocrine organ that secrets various hormones and cytokines for the regulation of energy homeostasis [2]. Skeletal muscles are a major site for the oxidation of both fatty acids and glucose (about 80% of insulin-stimulated glucose uptake). Furthermore, skeletal muscles contribute 30–40% of the resting metabolic rate in adults [3], which also plays a central role in the energy metabolism of the whole body. Additionally, the skeletal muscle represents one of the largest insulin-responsive tissues and utilizes a significant proportion of blood glucose during metabolism. Lu et al. [4] found butyrate supplementation to gestating sows and piglets induces oxidative-relative genes in muscle and adipose tissue and improves growth performance. Dietary supplementation of butyrate has been shown to prevent high-fat diet-induced obesity in mice [5] and in 3T3-L1 adipocytes [6]; however, other studies have shown that butyrate treatment attenuates lipolytic responses in rat primary adipocytes [7] and 3T3-L1 adipocytes [8] co-cultured with macrophages. These studies indicate that how butyrate affects the metabolism of adipose and muscle tissues remains controversial. Thus, understanding the molecular basis of adipose and muscle metabolic processes is necessary to prevent obesity and metabolic diseases.

The gut microbiome has emerged as a key regulator of the host energy metabolism, and its metabolites contribute to the metabolic function. Butyrate is a short-chain fatty acid (SCFA) that is mainly produced in the large intestine via the bacterial fermentation of non-digestible carbohydrates and can also be found in foods such as butter and cheese [9]. Butyrate has two major physiological functions in the regulation of the host metabolism. First, butyrate can be directly used as the energy source for colonic cells or can be directly involved in the glycolipid metabolism in peripheral tissues such as the liver [10,11]; second, butyrate can regulate many important metabolic activities in the body by activating the G protein-coupled receptor (FFAR2, FFAR3, and HCAR2) and by inhibiting histone deacetylase (HDAC) [12,13,14]. It has previously been reported that sodium butyrate induced metabolic adaptation in adipocytes via the upregulation of genes responsible for fatty acid oxidation as well as downregulation of genes involved in fatty acid synthesis in pigs [4]. Another study showed that 10 days of oral administration of sodium butyrate (SB) alleviates diet-induced obesity and insulin resistance in mice by activating the adiponectin-mediated pathway and stimulating the mitochondrial function in skeletal muscles [15]. Our previous study showed that short-term intravenous SB infusion modulates the hepatic lipid metabolism by increasing fatty acid transportation and cholesterol metabolism and decreasing fatty acid oxidation [16]. However, it remains unclear whether systemic butyrate administration differentially impacts the metabolism of different tissues.

RNA-Seq has become the favored technique due to its more quantitatively accurate measurement and since it allows to obtain absolute transcript abundance [17]. The mechanisms by which butyrate attenuates and ameliorates obesity and insulin resistance are not fully understood but clearly involve contributions from the skeletal muscle and adipose tissue. Although supplementation with butyrate can affect host energy homeostasis to improve the clinical efficacy of metabolic health, the information on the direct impact of butyrate on the muscle and adipose tissues is limited. The pig has been recognized as an ideal model for human nutrition research due to its similar anatomy and metabolism to humans [18]. Therefore, this study investigated the transcriptomic responses induced in muscle and adipose tissues via short-term intravenous infusion of SB in a pig model.

## 2. Materials and Methods

### 2.1. Ethics Statement

The trial was carried out under the supervision of the Animal Care and Use Committee of the Nanjing Agricultural University in Nanjing, Jiangsu, China [ethic code: SYXK (SU) 2017-0007].

### 2.2. Animals, Housing, Experimental Design, and Sampling

This study was approved by the Nanjing Agricultural University Animal Care and Use Committee (Nanjing, Jiangsu province, China). All animal procedures were previously described by Chen et al. [19]. In detail, 12 growing barrows (Duroc × Landrace × Large White) from the research pig farm were used. Each barrow was placed in an individual pen and received a commercial diet (metabolizable energy = 14.20 MJ/kg; crude protein = 16.8%) and water ad libitum. A medical polyethylene cannula (Braintree Scientific Inc, Braintree, MA, USA) was surgically installed into the internal jugular vein to enable the infusion of SB. After one week of recovery, pigs (BW = 23.70 ± 1.29 kg) were randomly allocated to SB and control (CO) groups (*n* = 6 per group) using a randomized complete block design. Pigs in the SB group were infused with 10 mL SB (200 mmol/L, pH 7.4, adjusted by NaOH) using syringes via the internal jugular vein each day, while pigs in the CO group were infused with the same volume of saline (0.9% NaCl, pH 7.4). The infusion from each pig was performed in 5 min. After a 7-day experimental period, all pigs were weighed and euthanized after 12 h of fasting. The 10th-rib *longissimus* *dorsi* muscle and dorsal subcutaneous adipose tissue were collected and preserved in liquid nitrogen for further transcriptomic analysis.

### 2.3. RNA Extraction and Purification

Total RNA of the muscle and adipose tissues was extracted using the RNeasy Mini Kit (Qiagen, GmBH, Germany), according to the manufacturer’s instructions. The RNA integrity number (RIN) was determined by an Agilent 2100 Bioanalyzer (Agilent Technologies, Santa Clara, CA, USA). The total RNA with RIN > 7.0 was further purified using the RNeasy Micro Kit (Qiagen, GmBH, Hilden, Germany) and RNase-Free DNase Set (Qiagen, GmBH, Hilden, Germany). RNA from each sample was used for library construction if it passed the quality control test.

### 2.4. Library Construction and Sequencing

Three biological replicates were randomly selected from each group for RNA-Seq, and library construction and sequencing were performed by Shanghai Biotechnology Corporation (Shanghai, China). Following purification, the mRNAs were fragmented into small pieces using divalent cations. The cleaved RNA fragments were copied into the first-strand cDNA, which was followed by second-strand cDNA synthesis. These cDNA fragments were then subjected to an end repair process, the addition of a single ‘A’ base, and ligation of adapters. The products were purified and PCR enriched to create the final cDNA library. Purified libraries were quantified by Qubit^®^ 2.0 fluorometer (Life Technologies, Grand Island, NY, USA) and validated by Agilent 2100 bioanalyzer (Agilent Technologies, CA, USA). Clusters were generated by cBot with the library diluted to 10 pmol/L and were then sequenced on the Illumina HiSeq 2500 platform (Illumina, San Diego, CA, USA), using paired-end sequencing. The raw RNA-seq reads were submitted to the Sequencing Read Archive database under the accession number GSE162636.

### 2.5. Data Analysis

After the sequencing was completed, the raw reads were stored in the FASTQ format. Then, the raw reads were cleaned by a short-reads pre-processing tool (FASTX-Tool kit, version 0.0.13) to filter out low-quality reads and trim adaptor sequences. The clean reads were mapped to the reference pig genome (Sus scrofa 11.1) by the mapping tool TopHat (version: 2.1.1) [20]. Each read allows multi hits ≤2, then Cufflinks (version: 2.2.1) was used for the quantitative analysis of mapping results [21]. The differentially expressed genes (DEGs) between SB and CO groups were qualified by the Cuffdiff program, using fragments per kilobase of exon model per million mapped reads (FPKM) [21]. *p* values were adjusted using the Benjamini and Hochberg method [22]. Corrected *p* value (*Q* value) <0.05 and fold change >1.2 or ≤0.83 were set as DEG threshold. In this study, the multi-omics data analysis tool OmicsBean (http://www.omicsbean.cn/, accessed on 6 June 2021), Geneforhealth, Shanghai, China) was employed to analyze the DEGs. Gene Ontology (GO) enrichment analysis, Kyoto Encyclopedia of Genes and Genomes (KEGG) pathway enrichment analysis of DEGs, and DEG-based protein-protein interaction (PPI) network were all integrated into this tool.

## 3. Results

### 3.1. Differentially Expressed Genes in Muscle and Adipose Tissues

**Figure 1:**
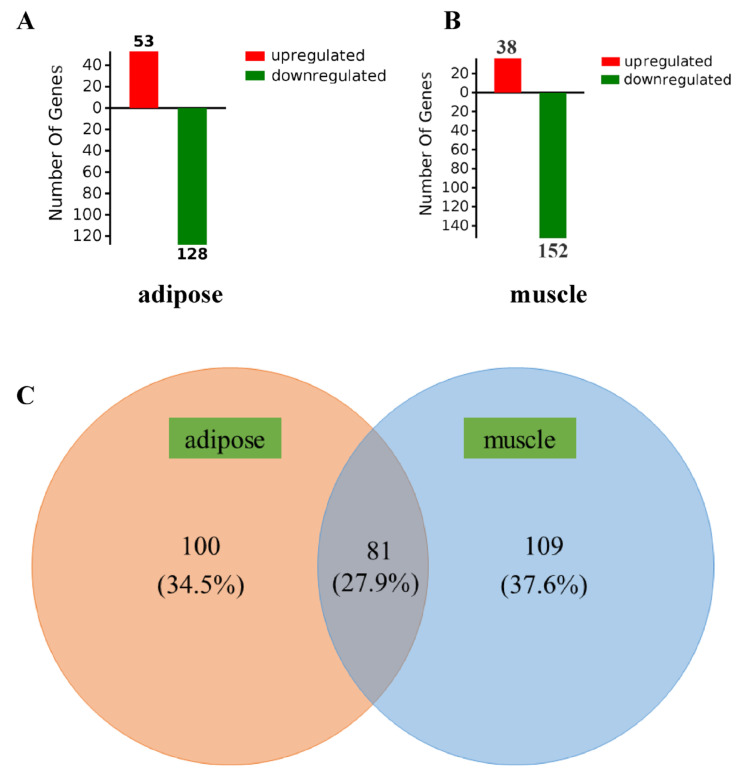
Numbers for common and specific differentially expressed genes (DEGs) between muscle and adipose tissues in response to intravenous infusion of sodium butyrate. (**A**,**B**) Red or green, respectively, represents the numbers of upregulated or downregulated genes. (**C**) Venn diagram of DEGs between different comparisons.

RNA-Seq analyses were performed in muscle and adipose tissues of pigs from the CO and SB groups. Differentially expressed genes were determined by using the same cut-offs (fold change > 1.2 or ≤ 0.83; *Q* value < 0.05) for both tissues. In the adipose tissue, a total of 181 DEGs were selected between CO and SB groups, 53 of which were upregulated and 128 were downregulated by SB treatment (Supplementary Table S1). In the muscle tissue, a total of 190 DEGs were selected between CO and SB groups, 38 of which were upregulated and 152 were downregulated by the SB treatment (Supplementary Table S2). In addition, 81 DEGs were identified in both muscle and adipose tissues, 109 DEGs were only found in muscle tissue, and 100 DEGs were only found in adipose tissue (Figure 1).

### 3.2. Functional Analysis of Differentially Expressed Genes in Both Muscle and Adipose Tissues

**Figure 2:**
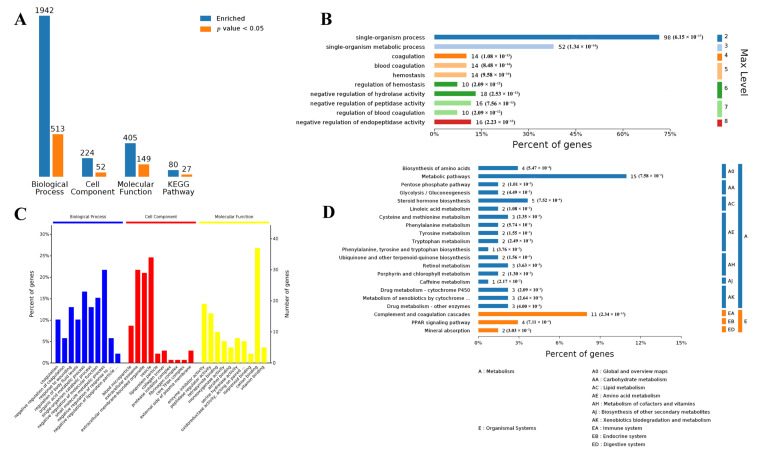
Bioinformatic analysis of DEGs in muscle tissue using four analysis categories: biological process (BP), cell component (CC), molecular function (MF), and Kyoto Encyclopedia of Genes and Genomes (KEGG) pathways. (**A**) Counts for each category represent the total associated terms in the database with the query protein list. Terms with *p* value < 0.05 are statistically significant. (**B**) BP hierarchy of DEGs, the number of involved genes, and corresponding *p* value in a specific biological process are shown on the right side of the column. (**C**) The ten most significantly enriched terms in level 4 of gene ontology hierarchy, information of percentage, and a number of involved genes in a term are shown in the left and right y-axes. (**D**) KEGG hierarchy of DEGs, the number of involved genes, and corresponding *p* value in a specific biological process are shown on the right side of the column.

To better understand the major metabolic pathways and physiological functions involved in DEGs induced by intravenous infusion of SB in both tissues, GO and KEGG pathway enrichment analyses of the DEGs were performed. Overall, the 190 DEGs in muscle tissue were annotated by biological process (BP) terms, and 513 terms were significantly enriched; DEGs were annotated by cell component (CC) terms, and 52 terms were significantly enriched; and DEGs were annotated by molecular function (MF) terms, and 27 terms were significantly enriched. Moreover, the top ten significantly enriched Gene Ontology terms (in level 4) are presented in Figure 2. Of the BP term, the small molecule metabolic process was the most represented term. Moreover, we observed that up to 52 DEGs were associated with a single-organism metabolic process. KEGG pathway enrichment analysis revealed that the pathway of metabolic pathways was significantly enriched (*p* value: 7.58 × 10^−3^), such as carbohydrate metabolism, lipid metabolism, amino acid metabolism, metabolism of cofactors and vitamins, biosynthesis of other secondary metabolites, and xenobiotics biodegradation and metabolism.

**Figure 3:**
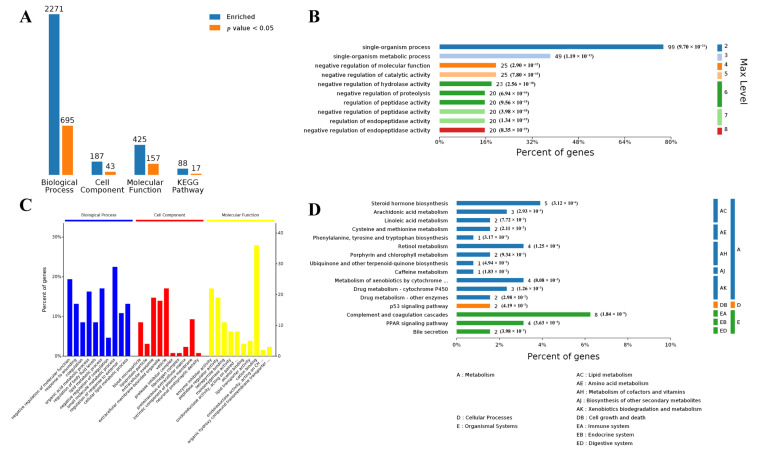
Bioinformatic analysis of DEGs in adipose tissue using four analysis categories: BP, CC, MF, and KEGG pathways. (**A**) Counts for each category represent the total associated terms in the database with the query protein list. Terms with *p* value < 0.05 are statistically significant. (**B**) BP hierarchy of DEGs, the number of involved genes, and corresponding *p* value in a specific biological process are shown on the right side of the column. (**C**) The ten most significantly enriched terms in level 4 of gene ontology hierarchy, information of percentage, and a number of involved genes in a term are shown in the left and right y-axes. (**D**) KEGG hierarchy of DEGs, the number of involved genes, and corresponding *p* value in a specific biological process are shown on the right side of the column.

In the adipose tissue, the 181 DEGs were annotated by BP terms, and 695 terms were significantly enriched; DEGs were annotated by CC terms, and 43 terms were significantly enriched; and DEGs were annotated by MF terms, and 17 terms were significantly enriched. Moreover, the top ten significantly enriched Gene Ontology terms (in level 4) are presented in Figure 3. Of the BP term, the small molecule metabolic process was also the most represented term. Moreover, we observed that up to 49 DEGs were associated with a single-organism metabolic process. KEGG pathway enrichment analysis revealed that the metabolism-related pathway was significantly enriched, including lipid metabolism and amino acid metabolism.

**Figure 4:**
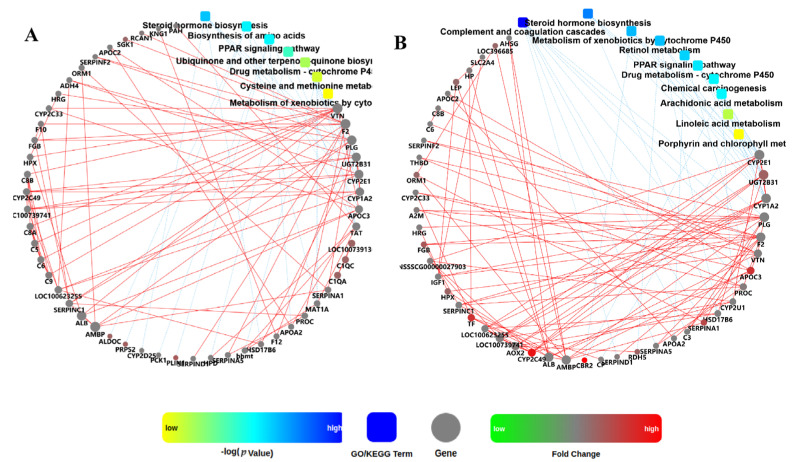
Protein-protein interaction (PPI) network for DEGs in the muscle (**A**) and adipose (**B**) tissues of pigs induced by the infusion of sodium butyrate. The PPI network is graphically displayed as nodes (genes or GO/KEGG terms) and edges (significant interactions among nodes). GO, Gene Ontology; KEGG, Kyoto Encyclopedia of Genes and Genomes.

Furthermore, to identify key DEGs induced by infusion of SB, a PPI network analysis based on DEGs in muscle and adipose tissues was performed (Figure 4). The network identified *LOC100739741*, *PLIN1*, and *PRPS2* as the top three circular nodes (genes) with the most edges in the muscle tissue, and *CBR2*, *CYP2C49*, and *APOC3* in the adipose tissue.

### 3.3. Comparison of DEGs Related to Metabolism in Muscle and Adipose Tissues

Genes related to metabolism and their major metabolic types in muscle and adipose tissues were summarized in Table 1. In muscle tissue, 11 genes were involved in carbohydrate metabolism. Among these, *PPP1R3B* and *ALDOC* genes involved in the carbohydrate catabolic process were upregulated; however, the *PCK1* gene involved in the carbohydrate biosynthetic process was downregulated. In adipose tissue, 12 genes were involved in carbohydrate metabolism. Among them, *LEP* and *IGF1* genes involved in the carbohydrate biosynthetic process were upregulated. In the muscle tissue, 28 genes were involved in the lipid metabolism, and 24 genes were downregulated. Among them, 8 genes (*CYP1A2, APOA2, CPS1, APOC2, CES3, LOC100737013, APOC3, PLIN1*) were involved in the lipid catabolic process, 7 genes (*PCK1, APOA2, APOC2, ACSM4, ETNK2, HSD17B13, APOC3, FASN*) were involved in lipid biosynthetic process, 2 genes (*APOA2, ARX*) were involved in lipid digestion, and only 1 gene (*APOA2*) was involved in lipid absorption. In the adipose tissue, 27 genes were involved in the lipid metabolism, and 22 genes were downregulated. Among them, 9 genes (*PNPLA3, CYP1A2, APOA2, CPS1, APOC2, LEP, LOC100737013, APOC3, LPL*) were involved in the lipid catabolic process, 11 genes (*PNPLA3, LOC100510957, APOA2, ACSM4, APOC2, DHCR24, STAR, LEP, C3, APOC3, LPL*) were involved in lipid biosynthetic process, 2 genes (*APOA2, LEP*) were involved in lipid digestion, and 2 genes (*APOA2, LEP*) were involved in lipid absorption. In the muscle tissue, 10 genes were involved in amino acid metabolism. Among these, 4 genes (*MAT1A, TDO2, TAT, HPD*) involved in the cellular amino acid catabolic process were downregulated; 2 genes (*CPS1, bhmt*) involved in the cellular amino acid biosynthetic process were downregulated. In adipose tissue, 10 genes were involved in amino acid metabolism. Among these, 1 gene (*TAT*) involved in the cellular amino acid catabolic process and 2 genes (*CPS1, bhmt*) involved in the cellular amino acid biosynthetic process were downregulated.

## 4. Discussion

As the main metabolite of gut microbial fermentation, butyrate has been considered an important mediator in regulating energy homeostasis. However, the metabolic mechanism of butyrate is still unclear. In the present study, the transcriptomic analysis was adopted to investigate the short-term effects of an intravenous SB infusion on the gene expression in muscle and adipose tissues of growing pigs. The transcriptome profile showed that short-term SB infusion significantly affected the amino acid metabolism in muscle tissue and the lipid metabolism in adipose tissue of growing pigs.

The results of KEGG enrichment showed that intravenous SB infusion significantly affected the amino acid metabolism pathway in muscle and adipose tissues of growing pigs in the short term. In the adipose tissue, DEGs were enriched in cysteine and methionine metabolism pathways and phenylalanine, tyrosine, and tryptophan biosynthesis pathways. Meanwhile, in the muscle, DEGs were enriched in the phenylalanine metabolism pathway, tyrosine metabolism pathway, tryptophan metabolism pathway, phenylalanine, tyrosine, and tryptophan biosynthesis pathways, and cysteine and methionine metabolism pathways. In addition, we found that most of the DEGs enriched in the amino acid metabolism were significantly downregulated, indicating that the intravenous SB infusion significantly downregulated the amino acid metabolism in muscle and adipose tissues of growing pigs in the short term.

At present, the mechanism of butyrate attenuating and ameliorating obesity and insulin resistance is not fully clear, but it clearly involves contributions from skeletal muscle and adipose tissue. Butyrate, as the energy substrate of the host, can be oxidized by fatty acids to CO_2_ or acetyl-CoA to synthesize lipids and glucose. In this study, carbohydrate metabolism-related pathways, such as the pentose phosphate pathway and glycolysis/gluconeogenesis pathway, were enriched in the skeletal muscle. The aldolase, fructose-bisphosphate C (*ALDOC*) gene, and the phosphoribosyl pyrophosphate synthetase 2 (*PRPS2*) gene in muscle were significantly upregulated; however, the phosphoenolpyrrolopyranoic acid carboxykinase 1 (*PCK1*) gene was significantly downregulated. As in the reverse pathway gluconeogenesis, ALDOC is a key enzyme in the fourth step of glycolysis. It plays a key role in ATP biosynthesis, due to it catalyzes the reversible conversion of fructose-1,6-diphosphate to glyceraldehyde-3-phosphate, or glyceraldehyde, and dihydroxyacetone phosphate by aldol cleavage [23,24]. SCFAs, such as butyrate and octanoate, can stimulate oxygen uptake in perfused liver and isolated hepatocytes to raise the energization of the mitochondria and to support ATP-dependent glucose generation [25,26,27]. In contrast to long-chain fatty acids (LCFAs) that are activated to acyl-CoAs in the cytosol and must be transferred to the mitochondrial interior via the carnitine shuttle, SCFAs permeate the inner mitochondrial membrane in the non-esterified form, activated by acyl-CoA synthetases, and are activated to their CoA-derivatives [28]. Compared with LCFAs, SCFAs (like octanoate) significantly raised the AMP level, which could inhibit the ATP-utilizing processes in the cell and stimulate those that produce ATP by activating AMP-dependent kinase (AMPK) [25,29]. Moreover, protein phosphatase 1 regulatory subunit 3B (PPP1R3B) belongs to a certain class of phosphatases which is known as protein serine/threonine phosphatases, which plays a crucial role in the regulation of the blood-glucose levels glycogen metabolism through ensuring the opposite regulation of glycogen breakdown and glycogen synthesis.

Leptin is a type of cytokine that is secreted by adipocytes, which can act on the central nervous system to maintain the energy balance by inhibiting animal appetite and promoting energy consumption [30]. Butyrate has been shown to regulate leptin expression through different signaling pathways in adipocytes [31]; our result also found that the *LEP* gene was upregulated in adipose tissue. Moreover, glucose metabolism-related genes, such as the insulin-like growth factor 1 (*IGF1*) gene and solute carrier family 2, facilitated the glucose transporter member 4 (*SLC2A4*) gene, were significantly downregulated in adipose, indicating that SB infusion decreased glucose utilization in adipose. Our results also indicated that the short-term infusion of SB in growing pigs upregulated glycogen breakdown and glycolytic pathways, downregulated the gluconeogenesis pathway in muscle, which was consistent with previous studies that butyrate significantly improved glucose tolerance, indicating that there were multiple mechanisms by which butyrate regulated glucose metabolism in the host system [5,32,33].

Studies showed that the reducing effect of butyrate on lipid content mainly occurred in BAT and to a lesser extent in the liver and muscle tissues [34]. In this study, the steroid hormone biosynthesis pathway and the linoleic acid metabolism pathway were enriched in the muscle tissue. Further, the key enzyme for the de novo synthesis of fatty acids [31], the fatty acid synthase (*FASN*) gene, was significantly upregulated in the muscle. The upregulated expression of the *FASN* gene suggested that the intravenous infusion of SB may promote fat deposition in muscle tissue. SB may exert this effect through butyrate-derived acetyl-CoA, which was consistent with the inhibition of glucose metabolism in muscle tissue. In addition, the steroid hormone biosynthesis pathway, linoleic acid metabolism pathway, and arachidonic acid metabolism were enriched in the adipose tissue.

Peroxisome proliferator-activated receptors (PPARs) are a group of nuclear receptor proteins that function as transcription factors in regulating the expression of genes and play an important role in regulating the metabolism of carbohydrates, lipid, and protein [35]. In the present study, the PPAR signaling pathway was enriched in muscle and adipose. The perilipin-1 (PLIN1) plays an important role in the regulation of basal and hormone-stimulated lipolysis [36], which was significantly upregulated in muscle. PLIN1 increased the formation of large lipid droplets, which suggested an increase in the synthesis of triglycerides [37], and a higher *PLIN1* expression was related to lower rates of lipolysis [38]. The angiopoietin-like 4 (*ANGPTL4*) gene was highly expressed in adipose tissue and was the target of PPARs. ANGPTL4 encoded a serum hormone protein or inhibited lipoprotein lipase (LPL) to participate in the regulation of lipid metabolism [39]. In our study, the downregulation of the *ANGPTL4* gene and the upregulation of the *LPL* gene in adipose tissue may activate lipolysis. Collectively, these results indicate that the effect of SB infusion on lipid metabolism increased de novo lipogenesis and decreasing lipolysis in muscle tissue, as well as increased lipolysis in adipose tissue. In addition, we found that SB treatment differently regulated the expression of genes related to carbohydrate, lipid, and amino acid metabolism in adipose and muscle tissues, which provided strategies for regulating meat quality by changing the metabolism of different tissues.

## 5. Conclusions

The present study showed differential transcriptomic responses in muscle and adipose tissues to short-term intravenous SB infusion. In muscle tissue, intravenous SB infusion affected the metabolism by downregulating the amino acid metabolism pathway, increasing the glycogen breakdown and glycolytic pathway, decreasing the gluconeogenesis pathway, increasing fatty acid synthesis, and decreasing the lipolysis pathway. In adipose tissue, intravenous SB infusion affected the metabolism by downregulating the amino acid metabolism pathway, decreasing the glucose utilization pathway, and increasing the lipolysis pathway. This study clarified the different transcriptomic responses to intravenous SB between muscle and adipose tissues in growing pigs. The results provided new evidence for the direct role of the SCFA butyrate on the regulation of energy metabolism; however, further studies are needed to evaluate the advantages or disadvantages of systemic butyrate on the metabolic health of animals or human beings.

## Figures and Tables

**Table 1:** Summary of DEGs associated with metabolism in muscle and adipose tissues.

Tissues	Genes ^1^	Major Metabolic Types
**muscle**	***PPP1R3B**, PCK1, APOA2, **ALDOC**, UGT2B31, LOC100623255, ANKH, MAT1A, **PRPS2**, ITIH2, ITIH1*	Carbohydrate metabolism
	***PLIN1**, ANKRD23, CES3, LOC10073013, HSD17B13, PCK1, CYP2D25, ETNK2, ACSM4, ADH4, CYP1A2, TTR, CYP2E1, APOC3, CPS1, GC, CYP2C33, CYP2C49, LOC100739741, APOA2, APOC2, **MYLIP**, ARX, **MLC1**, **ZNF703**, LOC100620829, LOC100623140, **FASN***	Lipid metabolism
	*PAH, HPD, TAT, TDO2, MAT1A, CPS1, bhmt, **SGK1**, HRG, APOA2*	Amino acid metabolism
**adipose**	***LEP**, C3, **IGF1**, **SLC2A4**, APOA2, **LPL**, UGT2B31, LOC100623255, UPP1, ITIH2, ITIH1, LBP*	Carbohydrate metabolism
	*APOC3, APOC2, **PNPLA3**, APOA2, **LPL**, LOC100737013, CYP2C49, **LEP**, LOC100510957, TTR, **DHCR24**, LOC100739741, CYP1A2, STAR, CYP2D25, CYP2E1, CPS1, CYP2C33, **GNLY**, ACSM4, C3, GC, LOC100620829, LBP, LOC100623140, SLCO2A1, ANGPTL4*	Lipid metabolism
	*bhmt, CPS1, TAT, DUOX2, TTR, **LEP**, **SFRP2**, PRDM8, HRG, APOA2*	Amino acid metabolism

^1^ Genes formatted in bold were upregulated, while the other genes were downregulated. DEGs, differentially expressed genes.

## Data Availability

Not applicable.

## Supplementary Materials

The following are available online at https://www.mdpi.com/article/10.3390/biology10060559/s1, Table S1: Differentially expressed genes in muscle tissue between CO and SB groups, Table S2: Differentially expressed genes in adipose tissue between CO and SB groups.
