# Supplementary material for: Transcriptomic Responses Induced in Muscle and Adipose Tissues of Growing Pigs by Intravenous Infusion of Sodium Butyrate
Source: Biology (Basel). 2021 Jun 20;10(6):559. doi: 10.3390/biology10060559 (PMC8234147; doi:10.3390/biology10060559)
Supplement: Supplementary file 1 [file biology-10-00559-s001.zip › biology-1195670-supplementary.pdf]

Table S1 Differentially expressed genes in muscle tissue between CO and SB groups

Gene id	Gene name	Log2(FC)	*P*-value	*Q*-value	Regulation
100037920	MT1D	-4.84	0.000	0.000	down
100037951	C9	-1.52	0.000	0.005	down
100037952	C6	-4.77	0.000	0.000	down
100037953	C8A	-5.23	0.000	0.000	down
100037954	C8B	-5.12	0.000	0.000	down
100037955	C8G	-1.97	0.000	0.000	down
100124383	CFB	-2.47	0.000	0.034	down
100125972	SERPINC1	-4.10	0.000	0.000	down
100126281	CYP3A46	-2.85	0.000	0.000	down
100134978	XIRP1	-1.55	0.000	0.005	down
100144442	F2	-4.14	0.000	0.000	down
100144468	CYP3A22	-4.26	0.000	0.001	down
100144531	PCK1	-2.72	0.000	0.000	down
100152095	HRG	-4.27	0.000	0.004	down
100152209	LOC100152209	-4.54	0.000	0.000	down
100152910	CYP1A2	-3.87	0.000	0.000	down
100153243	APOA2	-14.87	0.000	0.000	down
100153288	PZP	-13.21	0.000	0.000	down
100153302	SLC10A1	-4.49	0.000	0.000	down
100153386	SLA-DRB1	1.77	0.000	0.000	up
100153513	SERPINA5	-3.89	0.000	0.000	down
100153821	DDIT4	1.62	0.000	0.016	up
100153899	LOC100153899	-7.55	0.000	0.000	down
100154546	ABRA	-1.06	0.000	0.004	down
100155038	CPB2	-2.87	0.000	0.000	down
100155590	FRRS1L	2.51	0.000	0.016	up
100155644	PHIP	-0.60	0.000	0.020	down
100155795	MYLIP	1.13	0.000	0.033	up
100155919	UPB1	-3.73	0.000	0.000	down
100155945	SERPIND1	-4.81	0.000	0.000	down
100156325	LOC100156325	-5.51	0.000	0.000	down
100156500	DPYS	-4.80	0.000	0.000	down
100156540	RPP30	-0.73	0.000	0.021	down
100156922	MAT1A	-4.09	0.000	0.000	down
100157716	CPS1	-4.60	0.000	0.000	down
100157966	HABP2	-1.96	0.000	0.010	down
100169652	BMPR1A	-0.51	0.000	0.018	down
100170134	CD209	1.78	0.000	0.009	up
100301559	TDO2	-6.23	0.000	0.000	down
100381252	APOC2	-15.02	0.000	0.000	down
100510993	PRPS2	1.36	0.000	0.000	up
100511413	ASB5	-0.56	0.001	0.038	down

100511756	TAT	-4.04	0.000	0.000	down
100511802	GATD1	-0.83	0.000	0.032	down
100511902	RCAN1	-0.89	0.000	0.016	down
100512013	ALDOC	1.25	0.000	0.034	up
100512595	ACSM4	-3.60	0.000	0.000	down
100512615	ADH1C	-4.15	0.000	0.000	down
100513190	ASB15	-0.56	0.000	0.031	down
100513366	ADH4	-3.78	0.000	0.002	down
100513556	CAVIN3	1.12	0.000	0.007	up
100513784	SRA1	-5.52	0.000	0.000	down
100514063	UGT2B31	-5.88	0.000	0.000	down
100514354	FGB	-4.30	0.000	0.000	down
100515523	ALDOB	-5.42	0.000	0.000	down
100515741	LOC100515741	-6.38	0.000	0.000	down
100515857	LOC100515857	-3.19	0.000	0.000	down
100515931	PPP1R3B	1.22	0.000	0.002	up
100516396	CCL16	-2.89	0.000	0.000	down
100516651	FGL1	-3.18	0.000	0.000	down
100516980	A1BG	-3.96	0.000	0.000	down
100517431	HOXD10	3.27	0.000	0.000	up
100517580	TFR2	-3.74	0.000	0.000	down
100517609	FETUB	-4.41	0.000	0.000	down
100518846	C1QB	1.16	0.000	0.001	up
100518899	LECT2	-3.95	0.000	0.000	down
100519843	MLC1	1.89	0.001	0.049	up
100520813	AKR1D1	-3.65	0.000	0.000	down
100520915	COL11A2	3.22	0.000	0.022	up
100521900	PAH	-2.28	0.000	0.000	down
100522100	APOH	-4.40	0.000	0.000	down
100522234	CDC37L1	-0.54	0.000	0.030	down
100523371	APOB	-4.94	0.000	0.000	down
100523651	AKAP7	1.13	0.001	0.044	up
100524296	AGBL1	-1.19	0.000	0.000	down
100525011	ZNF703	1.18	0.000	0.007	up
100525254	SERPINF2	-1.98	0.000	0.005	down
100525457	ANKRD23	-0.74	0.000	0.011	down
100525860	CLTB	-0.55	0.000	0.006	down
100525894	DUSP26	-0.85	0.000	0.000	down
100620451	HOXA10	1.40	0.000	0.000	up
100620470	LOC100620470	-1.63	0.000	0.016	down
100620829	SLCO1B3	-3.17	0.000	0.000	down
100622319	FAM159B	-1.34	0.001	0.037	down
100622861	ETNK2	-3.05	0.000	0.002	down
100623255	LOC100623255	-4.61	0.000	0.000	down

100623917	SIM2	1.56	0.000	0.022	up
100623982	KBTBD8	-0.81	0.000	0.016	down
100624458	ANKH	-0.88	0.000	0.001	down
100625174	RETREG1	-1.16	0.000	0.012	down
100625739	SGK1	0.97	0.000	0.033	up
100626178	FGA	-4.58	0.000	0.000	down
100626664	YBEY	-0.51	0.000	0.021	down
100626686	LRMP	2.00	0.000	0.020	up
100626873	CES3	-1.85	0.000	0.017	down
100626886	HSD17B13	-2.44	0.000	0.013	down
100627139	ARX	-0.83	0.000	0.016	down
100628033	HOXC11	4.66	0.000	0.000	up
100736962	LOC100736962	-3.21	0.000	0.000	down
100738210	ENPP4	1.34	0.000	0.023	up
100739493	GTF2H5	-1.77	0.000	0.001	down
100739741	LOC100739741	-6.83	0.000	0.000	down
100739841	HGFAC	-4.25	0.000	0.000	down
102158419	SLC27A5	-1.44	0.000	0.007	down
102163953	GPA33	-1.37	0.000	0.000	down
102165584	LOC102165584	4.69	0.000	0.000	up
102166944	LOC102166944	-7.67	0.000	0.000	down
102167611	IGFN1	-0.79	0.000	0.020	down
106504545	LOC106504545	-4.28	0.000	0.000	down
106504547	LOC106504547	-4.18	0.000	0.002	down
106504800	LOC106504800	1.97	0.000	0.001	up
106506409	MAP6D1	1.66	0.000	0.009	up
106507969	LOC106507969	-4.77	0.000	0.000	down
106510389	LOC106510389	-0.60	0.000	0.018	down
110255234	LOC110255234	-1.89	0.000	0.011	down
110255453	LOC110255453	2.57	0.000	0.033	up
110255634	LOC110255634	-1.25	0.000	0.026	down
110255938	GCGR	-5.17	0.000	0.000	down
110256942	LOC110256942	-4.41	0.000	0.003	down
110257060	NIPA2	0.87	0.001	0.043	up
110257412	LOC110257412	4.32	0.000	0.000	up
110258309	LOC110258309	-3.52	0.000	0.000	down
110258312	LOC110258312	-4.51	0.000	0.000	down
110258925	LOC110258925	-14.53	0.000	0.000	down
110259259	SMTNL1	-0.79	0.000	0.006	down
110259298	LOC110259298	-0.77	0.000	0.005	down
110259691	LOC110259691	-1.22	0.000	0.006	down
110259958	LOC110259958	-0.99	0.000	0.021	down
110260032	LOC110260032	2.86	0.001	0.038	up
110260668	LOC110260668	7.88	0.000	0.000	up

110260683	METTL7B	-1.90	0.000	0.004	down
110261162	LOC110261162	-3.00	0.000	0.000	down
110261314	LOC110261314	-1.27	0.000	0.002	down
396568	KNG1	-4.69	0.000	0.000	down
396669	SPP2	-4.55	0.000	0.000	down
396684	LOC396684	-4.16	0.000	0.000	down
396685	LOC396685	-4.82	0.000	0.000	down
396686	SERPINA3-2	-4.20	0.000	0.000	down
396827	MT-2B	-2.50	0.000	0.000	down
396842	CRP	-2.52	0.000	0.000	down
396901	ORM1	-4.42	0.000	0.000	down
396921	APCS	-6.24	0.000	0.000	down
396937	ITIH2	-4.37	0.000	0.000	down
396954	PROC	-6.56	0.000	0.000	down
396960	ALB	-4.11	0.000	0.000	down
396963	ITIH1	-4.56	0.000	0.000	down
396996	TF	-3.90	0.000	0.000	down
396998	HPX	-3.58	0.000	0.000	down
397005	MYF6	-1.02	0.000	0.004	down
397049	SLC22A1	-12.25	0.000	0.000	down
397061	HP	-3.28	0.000	0.000	down
397124	RBP4	-1.43	0.000	0.008	down
397154	CHGB	2.67	0.000	0.009	up
397166	MS4A2	1.79	0.001	0.044	up
397192	VTN	-3.13	0.000	0.000	down
397371	BHMT	-6.20	0.000	0.000	down
397417	MT1A	-3.55	0.000	0.000	down
397419	TTR	-3.92	0.000	0.005	down
397443	HPD	-3.98	0.000	0.000	down
397474	F12	-2.56	0.000	0.000	down
397478	CES1	-1.93	0.000	0.000	down
397518	F9	-4.43	0.000	0.000	down
397534	SLCO1A2	3.54	0.000	0.000	up
397561	FASN	1.74	0.000	0.014	up
397585	AHSG	-4.47	0.000	0.000	down
397593	AMBP	-4.59	0.000	0.000	down
397660	ADIPOQ	1.78	0.000	0.002	up
397682	GSTA1	-5.29	0.000	0.000	down
397687	CYP2D25	-2.95	0.000	0.000	down
397688	SERPINA1	-1.53	0.000	0.000	down
403107	CYP2C33	-5.47	0.000	0.000	down
403108	CYP2C34	-5.72	0.000	0.000	down
403149	CYP2A19	-1.34	0.000	0.035	down
403164	FGG	-4.99	0.000	0.000	down

403215	CYP2C49	-5.89	0.000	0.000	down
403216	CYP2E1	-4.30	0.000	0.020	down
406187	APOC3	-4.47	0.000	0.000	down
406870	CP	-3.37	0.000	0.000	down
414437	C5	-2.58	0.000	0.000	down
445460	C1QC	1.12	0.000	0.009	up
445461	C1QA	1.16	0.000	0.028	up
448964	GC	-5.03	0.000	0.001	down
493185	IFRD1	-0.65	0.000	0.016	down
654411	PLIN1	1.56	0.001	0.044	up
733634	AKR1C1	-4.64	0.000	0.000	down
733640	PRKG1	-1.37	0.000	0.000	down
733660	PLG	-4.91	0.000	0.002	down
733662	F10	-1.85	0.000	0.001	down
780435	LOC780435	-1.93	0.000	0.036	down
gene2958	LOC407246	2.72	0.000	0.000	up

Corrected *P*-value (*Q*-value) < 0.05 and fold change > 1.2 or ≤ 0.83 were set as DEG threshold. DEGs, differentially expressed genes.

Table S2 Differentially expressed genes in adipose tissue between CO and SB groups

Gene id	Gene name	Log2(FC)	*P*-value	*Q*-value	Regulation
100037920	MT1D	-5.11	0.000	0.000	down
100037952	C6	-4.08	0.000	0.003	down
100037954	C8B	-6.25	0.000	0.000	down
100049690	PNPLA3	1.91	0.000	0.000	up
100125972	SERPINC1	-4.17	0.000	0.000	down
100144442	F2	-2.7	0.000	0.000	down
100144468	CYP3A22	-5.48	0.000	0.001	down
100144510	SLCO2A1	-1.17	0.000	0.015	down
100144585	HPCAL4	-2.59	0.000	0.030	down
100151902	PBXIP1	0.79	0.001	0.048	up
100152022	MTERF2	-2.87	0.000	0.007	down
100152095	HRG	-5.29	0.000	0.000	down
100152150	LOC100152150	-11.81	0.000	0.000	down
100152572	MAP3K20	-1.82	0.000	0.000	down
100152612	PPIF	0.84	0.001	0.048	up
100152741	TBX3	-1.16	0.000	0.012	down
100152785	DHRS7	0.89	0.000	0.028	up
100152910	CYP1A2	-4.38	0.000	0.002	down
100152997	GADD45G	1.59	0.000	0.005	up
100153243	APOA2	-5.67	0.000	0.000	down
100153288	PZP	-6.2	0.000	0.000	down
100153302	SLC10A1	-2.89	0.000	0.002	down
100153386	SLA-DRB1	1.31	0.000	0.002	up

100153513	SERPINA5	-5.25	0.000	0.000	down
100153570	ZIC1	1.72	0.000	0.011	up
100153899	LOC100153899	-3.08	0.000	0.008	down
100154866	KCNK5	2.16	0.000	0.003	up
100155010	PI16	-1.61	0.000	0.001	down
100155613	SVEP1	0.99	0.000	0.004	up
100155630	ZSWIM2	-2.42	0.000	0.000	down
100155930	ANXA8	-1.69	0.000	0.000	down
100155945	SERPIND1	-5.2	0.000	0.000	down
100156325	LOC100156325	-3.22	0.000	0.029	down
100157642	THBD	-1.12	0.000	0.012	down
100157716	CPS1	-4.82	0.000	0.000	down
100157783	NANOS1	-1.77	0.000	0.002	down
100157919	ARVCF	1.17	0.000	0.002	up
100307053	SELENOV	2.37	0.000	0.039	up
100327037	WNT4	-2.46	0.000	0.007	down
100381252	APOC2	-5.13	0.000	0.000	down
100510939	MATN4	-1.77	0.000	0.021	down
100510957	ACSM2B	-5.4	0.000	0.000	down
100511410	PDZRN3	1.17	0.000	0.017	up
100511727	EPHB6	1.4	0.000	0.004	up
100511756	TAT	-7.43	0.000	0.000	down
100512595	ACSM4	-5.37	0.000	0.000	down
100512615	ADH1C	-6.86	0.000	0.000	down
100513200	ANKRD34B	-2.11	0.000	0.028	down
100513770	SLC16A10	-1.01	0.000	0.006	down
100514063	UGT2B31	-4.66	0.000	0.000	down
100514243	PRDM8	-2.08	0.000	0.018	down
100514354	FGB	-3.57	0.000	0.000	down
100514801	TNS1	1.21	0.000	0.001	up
100514952	GALNT17	1.1	0.000	0.019	up
100515523	ALDOB	-6.84	0.000	0.000	down
100515741	LOC100515741	-5.93	0.000	0.000	down
100516027	SFRP2	1.21	0.000	0.012	up
100516785	DCP2	-1.9	0.000	0.000	down
100516839	WNT2	-2.27	0.000	0.004	down
100516911	USP44	-5.83	0.000	0.000	down
100516931	ARL4D	-1.88	0.000	0.006	down
100516980	A1BG	-3.55	0.000	0.000	down
100517145	LOC100517145	-2.53	0.000	0.033	down
100517431	HOXD10	2.48	0.000	0.040	up
100517580	TFR2	-3.28	0.000	0.008	down
100517586	RDH5	1.15	0.000	0.000	up
100518264	PIGY	-3.36	0.000	0.001	down

100518620	LOC100518620	1.11	0.000	0.005	up
100518899	LECT2	-3.74	0.000	0.001	down
100519066	HOXA9	1.4	0.000	0.002	up
100521236	HOXA11	6.18	0.000	0.001	up
100522100	APOH	-5.46	0.000	0.000	down
100523371	APOB	-5.98	0.000	0.000	down
100523701	AOX1	0.88	0.000	0.027	up
100524281	SCARA5	-1.43	0.000	0.028	down
100524958	DTX1	1.09	0.000	0.015	up
100525254	SERPINF2	-2.18	0.000	0.015	down
100525601	VASH2	-1.86	0.000	0.023	down
100525814	SIX2	1.69	0.000	0.028	up
100620451	HOXA10	2.96	0.000	0.000	up
100620470	LOC100620470	-1.35	0.000	0.020	down
100620623	SPOCK1	1.29	0.001	0.048	up
100620829	SLCO1B3	-2.86	0.000	0.003	down
100623255	LOC100623255	-6.47	0.000	0.000	down
100623423	GAP43	-2.61	0.000	0.007	down
100623504	LOC100623504	-11.03	0.000	0.000	down
100624042	SACS	3.55	0.000	0.000	up
100624404	SIK2	-1.28	0.000	0.003	down
100625277	UPP1	-2.25	0.000	0.001	down
100625649	ASGR1	-2.77	0.000	0.005	down
100625711	LOC100625711	-1.24	0.000	0.025	down
100626178	FGA	-5.26	0.000	0.000	down
100628034	ZNF276	1.13	0.000	0.021	up
100628125	HOXC10	1.8	0.000	0.007	up
100628197	DHCR24	0.81	0.000	0.025	up
100736894	TIA1	-0.85	0.000	0.042	down
100739741	LOC100739741	-5.89	0.000	0.000	down
100739753	TARDBP	-1.5	0.000	0.000	down
100739841	HGFAC	-3.12	0.000	0.000	down
102158081	NAT8L	1.3	0.000	0.004	up
102158419	SLC27A5	-2.71	0.000	0.007	down
102159780	LOC102159780	-2.31	0.000	0.019	down
102160006	LOC102160006	14.71	0.000	0.000	up
102160691	RXFP2	-1.84	0.000	0.030	down
102165231	LOC102165231	1.31	0.000	0.017	up
102167408	ADAMTS18	1.31	0.000	0.004	up
106504129	SLC6A6	-1.27	0.000	0.005	down
106504545	LOC106504545	-5.03	0.000	0.001	down
106504562	LOC106504562	-4.02	0.000	0.000	down
106505696	DZIP1L	0.8	0.000	0.036	up
106507757	LOC106507757	3.61	0.000	0.000	up

106508121	LOC106508121	5.43	0.000	0.000	up
106510070	YTHDF3	-1.18	0.000	0.009	down
110255234	LOC110255234	-1.89	0.000	0.005	down
110255237	LOC110255237	1.11	0.001	0.048	up
110256043	LOC110256043	1.13	0.000	0.019	up
110256069	DOC2B	-1.39	0.000	0.021	down
110256649	LOC110256649	2.62	0.000	0.022	up
110256777	LOC110256777	-1.45	0.000	0.024	down
110257075	LOC110257075	-5.54	0.000	0.000	down
110257412	LOC110257412	3.18	0.001	0.048	up
110258312	LOC110258312	-5.6	0.000	0.000	down
110259165	LOC110259165	-2.42	0.000	0.038	down
110259691	LOC110259691	-1.71	0.000	0.012	down
110259869	LOC110259869	2.13	0.001	0.048	up
110260668	LOC110260668	12.31	0.000	0.000	up
396554	COMP	1.65	0.000	0.002	up
396568	KNG1	-3.2	0.000	0.000	down
396597	STAR	-1.86	0.000	0.007	down
396598	GPX3	-1	0.000	0.014	down
396669	SPP2	-14.53	0.000	0.000	down
396684	LOC396684	-4.2	0.000	0.008	down
396685	LOC396685	-5.61	0.000	0.000	down
396686	SERPINA3-2	-4.69	0.000	0.000	down
396754	SLC2A4	0.94	0.000	0.012	up
396780	CBR2	-2.6	0.000	0.000	down
396832	LEP	2.63	0.000	0.000	up
396869	GNLY	1.29	0.000	0.016	up
396901	ORM1	-4.34	0.000	0.005	down
396921	APCS	-3.66	0.000	0.000	down
396937	ITIH2	-3.29	0.000	0.000	down
396954	PROC	-3.99	0.000	0.000	down
396960	ALB	-5.66	0.000	0.000	down
396963	ITIH1	-1.76	0.000	0.001	down
396996	TF	-3.13	0.000	0.000	down
396998	HPX	-3.06	0.000	0.000	down
397049	SLC22A1	-12.26	0.000	0.000	down
397060	DUOX2	-1.23	0.000	0.014	down
397061	HP	-2.67	0.000	0.000	down
397072	C3	-1.13	0.000	0.003	down
397192	VTN	-1.85	0.000	0.000	down
397303	LBP	-2.63	0.000	0.017	down
397371	BHMT	-6.97	0.000	0.000	down
397419	TTR	-5.27	0.000	0.000	down
397478	CES1	-1.7	0.000	0.006	down

397491	IGF1	0.93	0.000	0.033	up
397498	CHRM2	-2.18	0.000	0.014	down
397518	F9	-2.68	0.000	0.004	down
397534	SLCO1A2	2.23	0.000	0.001	up
397537	LPL	0.93	0.000	0.003	up
397585	AHSG	-6.13	0.000	0.000	down
397593	AMBP	-4.9	0.000	0.000	down
397628	ANGPTL4	-0.9	0.000	0.036	down
397687	CYP2D25	-2.54	0.000	0.001	down
397688	SERPINA1	-3.38	0.000	0.003	down
403107	CYP2C33	-4.7	0.000	0.000	down
403108	CYP2C34	-6.29	0.000	0.000	down
403164	FGG	-5.15	0.000	0.000	down
403166	A2M	-2.12	0.000	0.000	down
403215	CYP2C49	-13.81	0.000	0.000	down
403216	CYP2E1	-5.12	0.000	0.001	down
406187	APOC3	-4.57	0.000	0.000	down
406870	CP	-3.34	0.000	0.000	down
448964	GC	-6.91	0.000	0.000	down
492315	BMP7	-2.62	0.000	0.004	down
595111	KLF4	-1.05	0.000	0.025	down
733660	PLG	-5.35	0.000	0.001	down
733665	MMD	-1.25	0.000	0.003	down
733676	CSPG4	1.41	0.000	0.001	up
gene1617	LOC106509151	3.08	0.000	0.018	up
gene2958	LOC407246	2.09	0.000	0.000	up

Corrected *P*-value (*Q*-value) < 0.05 and fold change > 1.2 or ≤ 0.83 were set as DEG threshold.

DEGs, differentially expressed genes.
